# Editorial: Novel memristor-based devices and circuits for neuromorphic and AI applications

**DOI:** 10.3389/fnins.2023.1144906

**Published:** 2023-02-07

**Authors:** Heba Abunahla

**Affiliations:** Quantum and Computer Engineering Department, TU Delft, Delft, Netherlands

**Keywords:** memristor, switching, spiking, neurons, artificial intelligence

This Research Topic collects different contributions that highlight various aspects related to the deployment of MR devices for neuromorphic and AI applications. These contributions provide new insights toward utilizing emerging devices and approaches to mimic biological behavior of the brain.

The first article of this Topic (Poduval et al.), proposes a brain-inspired system that displays HDC memorization ability over a graph of relationships. The authors introduce GraphHD, hyper-dimensional memorization that displays graph-based information in high-dimensional space. This approach provides superior cognitive functions over the encoded memory graph, which allows the representation of complex graph-based data structure into high-dimensional space.

The contribution proposed by Fang, Liu et al. focuses on utilizing memristor devices in the leaky integrate-and-fire (LIF) spiking model, and introduces memristive-based LIF (MLIF). The authors prove the superiority of MLIF over LIF *via* comparing the firing patterns of both models. It's shown that MLIF can efficiently mimic the firing behavior of biological neurons. Experimental results show that a single memristive cell can emulate the synapse and demonstrate the function of integration and filtering of both the dendrite and soma. Thus, this work could provide a basic building block for reproducing the behaviors of biological neurons and building neural networks.

As an alternative approach, a memristive-based Izhikevich (MIZH) spiking neuron model is proposed in Fang, Duan et al.. The reported approach easily mimics the spiking and bursting patterns of distinct brain neurons. The MIZH neural network demonstrates both synchronous and asynchronous collective actions. The firing patterns of excitatory, inhibitory, and other neurons are also studied in this work using the MIZH model. Due to the inherent characteristics of memristor devices, MIZH will have a great impact on the design and implementation of artificial neural network models.

The authors in Fang, Tan et al. propose replacing the resistor with a memristor in the Resistor-Capacitor (RC) neuron circuit, and name the new circuit as Memristor-Capacitor (MC) circuit. It's shown that the charging and discharging time has been reduced using the MC circuit. Thus, due to the key advantages of memristor devices through the non-linear and non-volatile switching, this work proves the potential of utilizing memristor-based circuit to reproduce the behaviors of the biological neurons.

On a different study, the work reported in Garg et al. proposed a novel approach for brain-inspired unsupervised local learning. This study focuses on voltage-dependent-synaptic plasticity (VDSP). The authors show that the new methodology helps in reducing the number of updates compared to the standard spike timing dependent plasticity (STDP). Also, results prove that VDSP can be successfully deployed in pattern recognition applications.

We hope this Research Topic will be a great asset for the reader to enrich the knowledge and understanding of utilizing the unique and promising properties of emerging memristor devices for neuromorphic and AI applications.

## Author contributions

The author confirms being the sole contributor of this work and has approved it for publication.

